# Evolution of Exercise Training in Patients with Pulmonary Hypertension—A Comprehensive Review

**DOI:** 10.3390/healthcare14121796

**Published:** 2026-06-22

**Authors:** Ioannis Beis, Konstantina Dipla, Afroditi Boutou, Athanasios Zacharias, Athanasia Pataka, Evdokia Sourla, Andreas Zafeiridis, Georgia Pitsiou

**Affiliations:** 1 Respiratory Failure Unit, G. Papanikolaou Hospital, Aristotle University of Thessaloniki, 57010 Thessaloniki, Greece; giannisb1997@windowslive.com (I.B.); afboutou@auth.gr (A.B.); thanasiszachar@gmail.com (A.Z.); patakath@auth.gr (A.P.); evisou@yahoo.gr (E.S.); zafeirid@phed-sr.auth.gr (A.Z.); gpitsiou@auth.gr (G.P.); 2 Laboratory of Exercise Physiology and Biochemistry, Department of Physical Education and Sports Science at Serres, Aristotle University of Thessaloniki, 62100 Serres, Greece; 3 School of Life and Health Sciences, University of Nicosia, Nicosia CY-2417, Cyprus

**Keywords:** pulmonary hypertension, exercise, pulmonary rehabilitation, exercise training, exercise capacity, cardiorespiratory fitness, right heart

## Abstract

Pulmonary hypertension (PH) is a progressive, multifactorial syndrome characterized by elevated pulmonary arterial pressure and right heart dysfunction, associated with significant morbidity, impaired quality of life, and poor prognosis. Advances in classification, hemodynamic definitions, and targeted pharmacotherapies have improved understanding and management, yet therapeutic challenges persist across the five World Health Organization groups of PH. Historically, exercise was discouraged due to concerns about adverse hemodynamic effects, but growing evidence has suggested that structured, supervised training is safe and beneficial. Randomized trials and meta-analyses show improvements in six-minute walk distance, peak oxygen uptake, right ventricular function, ventilatory efficiency, and health-related quality of life, with a low incidence of adverse events. Physiological adaptations include favorable cardiac remodeling, enhanced endothelial function, improved skeletal and respiratory muscle performance, and improved neurohormonal activity. Despite this evidence, barriers such as patient fears, limited clinical expertise, and restricted access to specialized rehabilitation programs hinder widespread implementation. Current guidelines recommend supervised exercise as part of pulmonary rehabilitation for patients with stable PH, supporting its role as an adjunct to pharmacotherapy. This descriptive review briefly summarizes the pathophysiology of PH, phenotype-related differences and current therapeutic approaches, and the beneficial adaptations to exercise training, with the aim of informing exercise specialists and supporting safer, more effective integration of exercise-based rehabilitation into patient care.

## 1. Introduction

Pulmonary hypertension (PH) is a complex clinical syndrome affecting the pulmonary vasculature and progressively impairing right heart function [1]. It has a broad etiology, including idiopathic, heritable, drug-induced, and secondary forms linked to left heart disease, lung disease, chronic thromboembolism, or systemic disorders. Though PH is rare, it carries a significant disease burden due to its progressive nature, diagnostic challenges, and limited therapies [2]. Its symptoms, such as exertional dyspnea, fatigue, syncope, and edema, are often nonspecific, delaying diagnosis. Progressively, there is a decline in functional capacity, deconditioning, and psychological issues such as anxiety and depression, resulting in impaired quality of life and poor prognosis [3]. In addition, PH contributes to significant social and economic burden, due to patients’ frequent hospitalizations, specialized care, and need of long-term treatment [4]. These challenges highlight the need for a multidisciplinary approach combining medical, psychological, and rehabilitative care [2,3,4].

Exercise training is widely recognized as a cornerstone therapeutic intervention for chronic diseases [5]. Robust evidence from large-scale meta-analyses and clinical guidelines has established that exercise training improves cardiorespiratory fitness, muscle strength, and overall functional status in patients with chronic illnesses [6]. In conditions such as chronic obstructive pulmonary disease (COPD) and interstitial lung disease (ILD), exercise training–pulmonary rehabilitation, receives a strong recommendation in guidelines by leading organizations such as the American Thoracic Society (ATS) and the European Respiratory Society (ERS), as substantial improvements in symptoms, quality of life, and reduced hospitalizations have been reported in several studies [7]. In contrast, for patients with PH, the historical approach was one of caution, with patients being advised to restrict physical activity due to fears of adverse events such as syncope, right ventricular decompensation, and sudden cardiac death [8]. Over the last two decades, however, growing evidence has challenged this paradigm, demonstrating that carefully prescribed and supervised exercise training can be both safe and beneficial in clinically stable PH patients [9]. This shift from exercise restriction toward exercise prescription has gradually positioned pulmonary rehabilitation as an emerging adjunctive therapy within comprehensive PH management [1,7]. The latest ATS guidelines give a conditional recommendation for pulmonary rehabilitation in PH, acknowledging promising but still developing evidence [7]. Physiological benefits of exercise in pulmonary disease include enhanced cardiovascular function, muscle strength oxygen efficiency, and brain oxygenation, resulting in reduced ventilatory demand and improved exercise tolerance [10]. In PH, these adaptations can delay the appearance of dyspnea, improve right ventricular function, and enhance quality of life, with additional psychological benefits like reduced anxiety and depression [11,12].

In light of the evolving guideline recommendations on exercise training in PH, this review briefly presents the characteristics and phenotypes of PH, outlines current treatment options, and provides an overview of pulmonary rehabilitation with a focus in exercise training in PH, highlighting the emerging role of pulmonary rehabilitation as an adjunct to optimized medical therapy in the comprehensive management of PH. The main novelty of this review is its phenotype-specific perspective. Specifically, this review aims to link the disease phenotype with implications for exercise prescription and to facilitate individualized, exercise rehabilitation strategies in clinical practice. Specifically, the aims of this review were to (i) present current treatment approaches and exercise limitations across the different pulmonary hypertension subtypes, and (ii) discuss considerations for exercise program design based on the available evidence, while highlighting current gaps in exercise prescription and rehabilitation research.

For this review a literature search was conducted in PubMed and Cochrane Library databases using the following keywords: “pulmonary hypertension,” “pulmonary arterial hypertension,” “chronic thromboembolic pulmonary hypertension,” “exercise”, “exercise training,” “pulmonary rehabilitation” “rehabilitation”. Both “All Fields” terms and MeSH (Medical Subject Headings) terms were included. The search was expanded using “OR” and “AND”. Only studies in adult patients with PH were included. The search included studies published up to January 2026.

## 2. Classification and Epidemiology of Pulmonary Hypertension

### 2.1. World Health Organization Classification of PH

The classification of PH has evolved in parallel with the understanding of its diverse etiologies and clinical presentations. Initially described as “primary pulmonary hypertension” in early clinical literature, PH was considered a rare, idiopathic condition [13]. However, understanding of its association with various cardiopulmonary and systemic diseases led the World Health Organization to propose a structured classification system, with the latest revision published in 2022 [1].

PH is currently classified into five groups according to WHO. Each group has unique epidemiological patterns, risk factors, and clinical implications. The main etiologies of PH are summarized in Table 1.

#### 2.1.1. Group 1: Pulmonary Arterial Hypertension (PAH)

Pulmonary arterial hypertension (PAH), classified as Group 1 PH, is a rare but serious vascular disease that includes idiopathic, heritable, and drug- or toxin-induced PAH, as well as PAH associated with conditions such as connective tissue disease, HIV infection, portal hypertension, congenital heart disease, and schistosomiasis [1]. It is characterized by remodeling of small pulmonary arteries, leading to increased pulmonary vascular resistance [14]. The disease predominantly affects women and is most often diagnosed in middle-aged and older adults [15,16,17]. The Global Burden of Disease Study 2021, estimated a prevalence of about 2.28 cases per 100,000 population worldwide [2]. Although advances in diagnosis and treatment have improved outcomes, PAH remains associated with substantial morbidity and mortality.

#### 2.1.2. Group 2: Pulmonary Hypertension Due to Left Heart Disease

Group 2 PH results from left ventricular systolic or diastolic dysfunction, or valvular heart disease, leading to elevated pulmonary pressure secondary to increased left-sided filling pressures. It is the most common form of PH, accounting for 50–85% of all cases, with prevalence increasing with age and comorbidities [18]. PH is frequently observed in severe aortic or mitral valve disease, and may persist after intervention, which is associated with poorer outcomes [19,20]. Recent guidelines distinguish isolated post-capillary PH from combined pre- and post-capillary PH, the latter associated with higher mortality and complex management [21].

#### 2.1.3. Group 3: PH Due to Lung Diseases and/or Hypoxia

Group 3 PH includes PH associated with chronic lung diseases (COPD, ILD, sleep-disordered breathing), and other causes of chronic hypoxia. Hypoxic vasoconstriction and lung vasculature remodeling contribute to pressure elevation [22]. Its prevalence and severity vary substantially by the underlying disease. In advanced COPD, mild PH is relatively common, yet severe PH is rare. However, even mild to moderate PH in this population is associated with reduced exercise tolerance and increased mortality [23]. In ILD, the presence of PH is even more frequent (prevalence rates 34–36%). In ILD, PH correlates strongly with impaired gas exchange, reduced quality of life, and worse prognosis [24]. Obstructive sleep apnea is another significant risk factor (reported prevalence 17–70%) [25]. High-altitude PH affects certain populations living above 2500 m, where chronic hypoxia can induce pulmonary vascular remodeling and PH [26]. Although prevalence and severity of Group 3 PH vary by underlying condition, age, and cardiopulmonary comorbidities, its presence is generally associated with reduced exercise capacity, poorer quality of life, and worse clinical outcomes [23,24,25,26,27].

#### 2.1.4. Group 4: Chronic Thromboembolic Pulmonary Hypertension

Chronic thromboembolic pulmonary hypertension (CTEPH) results from unresolved thromboembolic obstruction in the pulmonary arteries, after acute pulmonary embolism. CTEPH is rare (prevalence 26–38 per million adults) [17]. Among survivors of acute PE, 0.5–3% develop CTEPH over several years, with higher risk in those with recurrent events, certain thrombophilic conditions, or delayed diagnosis [28,29]. CTEPH is clinically important as it is the only potentially curable form of PH with surgical or catheter-based interventions available [30].

#### 2.1.5. Group 5: PH with Unclear or Multifactorial Mechanisms

Group 5 PH comprises several rare conditions with complex or unclear mechanisms: hematologic disorders (e.g., chronic myeloproliferative diseases and sickle cell disease), metabolic syndromes, chronic kidney disease, fibrosing mediastinitis, sarcoidosis, and certain tumoral microangiopathies [31]. Epidemiological data are limited due to the diversity and rarity of these disorders [31,32,33,34,35,36,37,38]. Although uncommon overall, Group 5 PH remains clinically significant in affected subgroups, and requires individualized diagnosis and approach due to its multifactorial pathogenesis.

### 2.2. Hemodynamic Definition

In addition to clinical classification, PH is defined by hemodynamic criteria, and exertional PH is recognized as a clinical entity [1,39]. According to the 2022 ESC/ERS Guidelines [1], PH is diagnosed when the mean pulmonary artery pressure (mPAP) exceeds 20 mmHg at rest, as measured by right heart catheterization. Further subclassification is based on pulmonary artery wedge pressure (PAWP) and pulmonary vascular resistance (PVR), as presented in Table 2. Each group has distinct causes, management approaches, and prognostic implications. However, overlap between groups is common, and accurate classification often requires comprehensive multidisciplinary assessment. 

## 3. Pathophysiology of Pulmonary Hypertension

The pathophysiological mechanisms vary significantly across the major PH groups, reflecting the distinct underlying diseases and processes.

### 3.1. Pathophysiology of Pulmonary Arterial Hypertension

PAH is characterized by elevated pulmonary arterial pressure, primarily due to structural and functional changes in the small pulmonary vessels [40]. Its pathogenesis is driven by pulmonary vascular remodeling involving all vessel wall layers, endothelial dysfunction, and an imbalance of vasoactive mediators, such as increased endothelin-1 and reduced nitric oxide and prostacyclin [41,42]. These alterations promote vasoconstriction, inflammation, thrombosis, smooth muscle proliferation. Additional contributors include dysregulated BMPR2 signaling, activation of serotonin and Rho-associated kinase pathways, and inflammatory cell infiltration [43,44]. Additionally, mitochondrial dysfunction, both a cause and a consequence of excessive oxidative stress, contributes to metabolic reprogramming in pulmonary vascular cells, resulting in a shift from oxidative phosphorylation to glycolytic pathways (“Warburg effect”) in endothelial and smooth muscle cells, contributing to pathological cell proliferation and apoptosis [45].

Histologically, PAH is characterized by medial hypertrophy, intimal fibrosis, and plexiform lesions, representing disorganized endothelial proliferation that can obstruct vessels [46]. Loss of distal pulmonary arteries and microvascular rarefaction worsen the pressure load [47]. Over time, right ventricular hypertrophy compensates for increased afterload, but chronic stress leads to RV dilation, dysfunction, and ultimately right heart failure, the primary cause of death in PAH.

### 3.2. Pathophysiology of Pulmonary Hypertension Due to Left Heart Disease

The most common form of PH results from chronically elevated left atrial pressures transmitted backward into the pulmonary circulation [48]. Initially, this leads to isolated post-capillary PH, characterized by passive venous congestion without significant pulmonary vascular remodeling. However, in some patients, sustained high pressures, combined with endothelial dysfunction, shear stress, and chronic inflammation induced arteriolar remodeling, smooth muscle hypertrophy, and increased PVR, features of combined pre- and post-capillary PH [49,50]. Comorbidities like diabetes, obesity, and atrial fibrillation further exacerbate vascular dysfunction [51]. The right ventricle in Group 2 PH struggles with increased afterload, and once decompensation occurs, outcomes worsen dramatically.

### 3.3. Pathophysiology of Pulmonary Hypertension Due to Lung Diseases and/or Hypoxia

Pulmonary hypertension associated with chronic lung diseases results from an interplay of chronic hypoxia, inflammation, pulmonary vascular remodeling, loss of vessels [52,53,54]. Persistent alveolar hypoxia triggers hypoxic pulmonary vasoconstriction, intended to optimize gas exchange. Chronically, however, this leads to structural remodeling of the pulmonary arteries [55]. Although its severity often parallels the extent of underlying lung damage, PH may also develop independently of hypoxia, reflecting complex vascular pathobiology [53]. PH in this group worsens exercise capacity and survival, particularly in patients with combined pulmonary fibrosis and emphysema [56].

### 3.4. Pathophysiology of Chronic Thromboembolic Pulmonary Hypertension

CTEPH results from incomplete resolution of pulmonary emboli, leading to organized fibrotic thrombi that obstruct pulmonary arteries [57]. Mechanical obstruction causes redistribution of blood flow, increased shear stress, and progressive vascular remodeling in unobstructed areas [58]. Histopathologically, CTEPH features fibrotic bands, webs, and secondary small vessel arteriopathy resembling that seen in PAH [59]. Beyond thrombotic occlusion, endothelial dysfunction, chronic inflammation, impaired fibrinolysis, and abnormal angiogenesis contribute to the development and progression of disease [60].

## 4. Current Management Strategies and Emerging Therapies for Pulmonary Hypertension

Management of PAH has evolved with improved understanding of disease mechanisms and clinical evidence, with current strategies emphasizing early diagnosis, risk stratification, and targeted therapy. Risk assessment integrates symptoms, exercise capacity, right ventricular function, and biomarkers such as NT-proBNP to guide treatment intensity [60]. Therapy targets three main pathways, prostacyclin, endothelin, and nitric oxide, using endothelin receptor antagonists, phosphodiesterase-5 inhibitors, soluble guanylate cyclase stimulators, and prostacyclin-based therapies [61,62,63,64,65,66]. Novel agents such as sotatercept and therapies targeting the TGF-β/BMP pathway aim to further modify disease progression [67,68,69,70,71,72,73].

Management of non-PAH PH focuses on the underlying cause, including guideline-directed heart failure therapy in Group 2, oxygen and disease-specific treatment in Group 3, and interventions such as pulmonary endarterectomy or balloon pulmonary angioplasty in CTEPH [74,75,76,77,78,79].

Non-pharmacological care, including pulmonary rehabilitation, education, psychosocial support, and preventive measures, plays an important adjunctive role. Exercise-based rehabilitation improves functional capacity and quality of life in stable patients but should complement rather than replace pharmacological therapy within a multidisciplinary approach [1,7,80]. The role of exercise in PH and the mechanisms responsible for the beneficial adaptations in response to exercise training are discussed below.

## 5. Effectiveness of Exercise Training in Pulmonary Arterial Hypertension

### 5.1. Evolving Clinical Perspectives on Exercise in Pulmonary Hypertension

Traditionally, patients with PH, particularly those with PAH, were advised to avoid physical activity. This caution was based on concerns that exercise could worsen right ventricular dysfunction and provoke hemodynamic instability. The 2009 ACC/AHA expert consensus considered exercise training and PR was contraindicated due to fears of sudden cardiac death and right heart decompensation [81]. Similarly, early definitions of exercise-induced PH suggested that exceeding certain hemodynamic thresholds during exertion caused pathological responses, reinforcing the belief that exercise posed risks [82]. However, this conservative approach began to change later, when early pilot studies suggested that structured, low-to-moderate intensity aerobic training under medical supervision could be both safe and beneficial for patients with stable PH [83]. These studies showed improvements in exercise capacity, symptoms, and quality of life, challenging long-held concerns. This evolving evidence was reflected in the 2009 ESC/ERS guidelines, which cautiously recommended supervised rehabilitation for physically deconditioned PAH patients in controlled environments [8]. These guidelines emphasized that while patients should remain active within their symptom thresholds, activities causing severe dyspnea, chest pain, or dizziness should be avoided. Mild dyspnea was considered acceptable, whereas peripheral muscle wasting remained inadequately addressed, despite sarcopenia being a significant health concern.

### 5.2. Key Clinical Trials and Observational Studies in Pulmonary Hypertension

A landmark randomized controlled trial by Mereles et al. evaluated a 15-week supervised exercise program in PAH patients and showed significant improvements in six-minute walk distance (6MWD), peak oxygen uptake (VO2peak), and health-related quality of life (HRQoL), with no serious adverse events [83]. Furthermore, Grünig et al. conducted a prospective study in a larger, more heterogeneous PH cohort, confirming the safety and efficacy of a similar 15-week program. The intervention led to significant improvements in 6MWD, VO2peak, oxygen pulse, WHO functional class, and several domains of HRQoL, even in patients with WHO functional class IV [84].

Bussotti et al. reported that a 4-week outpatient program incorporating aerobic and resistance training, inspiratory muscle training, and psychological support led to improvements in VO2peak, 6MWD, and mental health parameters such as anxiety and depression, as assessed by the Hospital Anxiety and Depression Scale (HADS) and EuroQoL-5D (EQ-5D) questionnaire [85]. Similarly, Nagel et al. studied 35 patients with inoperable or residual CTEPH who completed a 3-week inpatient program followed by 15 weeks of home-based training and showed significant gains in functional capacity, quality of life, and NT-proBNP levels [86]. A meta-analysis of 17 studies involving 651 PH patients reported a significant increase in 6MWD and in VO2peak, along with significant quality-of-life improvements and a low incidence of adverse events [87]. It should be noticed that most available evidence is derived from studies in PAH and/or CTEPH, while the remaining PH groups have been largely understudied.

### 5.3. Effectiveness of Exercise Training in Chronic Lung and Heart Diseases

In chronic lung diseases, pulmonary rehabilitation with aerobic and resistance training improves dyspnea, exercise capacity, and quality of life in COPD [86], enhances functional and psychological outcomes in ILD [88], and supports airway clearance and lung function in cystic fibrosis [89], with shared mechanisms including peripheral muscle deconditioning, ventilatory inefficiency, and systemic inflammation. Similarly, in chronic heart diseases, particularly heart failure with reduced ejection fraction, structured exercise training improves VO2peak, functional capacity, and quality of life, with additional benefits in hospitalization and mortality risk, mediated through improved endothelial function, autonomic balance, and skeletal muscle efficiency [90,91,92,93,94]. Benefits are also observed in heart failure with preserved ejection fraction and ischemic heart disease, where cardiac rehabilitation is guideline-recommended and associated with improved clinical outcomes [92,93,95]. Across both disease groups, exercise consistently enhances cardiorespiratory reserve, skeletal muscle function, and physical activity levels, leading to better exercise tolerance and quality of life [96,97]. These findings support the relevance of exercise-based interventions as a model for pulmonary hypertension; however, it should be noted that several of the described mechanisms are extrapolated from the other chronic cardiopulmonary conditions, as PH-specific mechanistic data are limited. Therefore, evidence from chronic lung and heart diseases should be considered supportive and translational rather than equivalent to PH-specific evidence.

## 6. Mechanisms of Physiological Adaptations Induced by Exercise Training in Patients with Pulmonary Hypertension

### 6.1. Right Ventricular Remodeling and Hemodynamics

A central goal of exercise training in PH is to preserve and improve RV function, which subsequently determines prognosis. Limited clinical data suggest that exercise training in patients with PH may induce beneficial changes in RV structure and function, with some studies reporting improvements in stroke volume, cardiac output, and markers of reverse remodeling following rehabilitation programs [98]. Preclinical evidence further supports these observations, as animal studies have shown attenuation of RV hypertrophy, fibrosis, and diastolic dysfunction after exercise training in monocrotaline-induced PH models [99]. In addition, in rats with monocrotaline-induced PH, low- to moderate-intensity resistance training induced beneficial structural and functional adaptations in the lungs, right ventricle, and skeletal muscle [100]. These experimental data suggest that regular exercise may reduce RV afterload, possibly by reducing pulmonary vascular resistance and enhancing ventricular–arterial coupling, a dynamic relationship shown to be impaired in pulmonary arterial hypertension during exercise [101]. Although these findings are promising, the extent to which exercise training can induce clinically meaningful RV reverse remodeling in humans remains incompletely established.

### 6.2. Vascular and Endothelial Adaptations

Evidence regarding vascular and endothelial adaptations to exercise training in pulmonary hypertension derives from a combination of human studies, preclinical models, and indirect evidence from other cardiovascular diseases. Exercise promotes beneficial vascular remodeling driven by increased shear stress, which stimulates upregulation of endothelial nitric oxide synthase and enhances nitric oxide bioavailability, contributing to improved vascular function [102].

Preclinical experimental data suggest that exercise may have a direct effect on pulmonary vascular remodeling. In more detail, animal models of hypoxia-induced PH undergoing swimming training have shown reductions in medial hypertrophy, pulmonary arterial pressure, and inflammatory markers, suggesting modulation of pulmonary vascular pathology [103]. In addition, in rats with monocrotaline-induced PH, resistance training has been shown to attenuate increases in pulmonary vascular resistance and improve markers of oxidative stress, suggesting beneficial effects on pulmonary vascular function [100]. These adaptations were associated with improved survival and physical effort tolerance (i.e., maximum carrying load) in the experimental animal.

Clinical evidence in patients with PH remains more limited. Supervised aerobic exercise has been shown to improve pulmonary hemodynamics and vascular function in patients with PH [104]. Further support for the vascular benefits of exercise comes from non-PH populations, in whom regular aerobic exercise improves endothelial function, increases nitric oxide bioavailability, reduces oxidative stress, and enhances arterial compliance [105]. Although the available evidence is promising, the specific impact of exercise training on pulmonary vascular remodeling and its potential reversibility in humans with PH remains incompletely understood and warrants further investigation.

### 6.3. Neurohormonal and Autonomic Regulation

Pulmonary hypertension is associated with increased sympathetic nervous system activity and neurohormonal dysregulation, both of which contribute to sustained vasoconstriction, right ventricular dysfunction, and systemic complications [106]. Specifically, chronic activation of the renin–angiotensin–aldosterone system (RAAS) has been documented in PAH, promoting vascular remodeling and further exacerbating pulmonary vascular resistance [107].

Direct evidence regarding exercise-induced neurohormonal adaptations in PH patients remains limited. Current understanding is largely derived from indirect evidence in heart failure and COPD populations, where regular exercise has been shown to improve autonomic balance by reducing sympathetic activity, lowering resting heart rate, and modulating neurohormonal pathways [108]. Exercise may further influence the RAAS and inflammatory cytokine signaling, contributing to improved cardiovascular regulation [109]. Similar mechanisms may contribute to the beneficial effects observed in PH patients, although dedicated mechanistic studies are needed to confirm these adaptations in this population.

### 6.4. Skeletal and Respiratory Muscle Adaptation

Among the proposed mechanisms underlying exercise benefits in PH, skeletal and respiratory muscle adaptations are supported by evidence from both human studies and experimental models. PAH patients exhibit peripheral myopathy characterized by reduced oxidative fiber proportion, mitochondrial dysfunction or impaired oxygen delivery to mitochondria possibly associated with decreased capillarity, and reduced strength in both skeletal and respiratory muscles [110,111]. Although the mechanisms by which exercise training improves muscle function in PH have not been fully established [112], evidence in non-PH populations suggests that exercise training promotes mitochondrial biogenesis [113]. Newly formed mitochondria fuse with existing mitochondria, facilitating the exchange of metabolites, proteins, and mtDNA, thereby enhancing ATP production and oxidative metabolism. Exercise also promotes mitochondrial quality control through fission and mitophagy, processes associated with reduced cellular stress and inflammation [111,113]. In addition, exerkines released during exercise may contribute to systemic adaptations affecting the vascular system and other organs [114]. In addition, skeletal muscle-derived VEGF and angiopoietin-1 promote angiogenesis and neovascularization, potentially contributing to improved endothelial function and tissue perfusion. In non-PH populations, these adaptations have been associated with improved oxygen utilization during exercise and may provide a potential explanation for the gains in 6MWD and VO2peak observed following rehabilitation programs in PH. Additionally, inspiratory muscle training has been shown to improve respiratory muscle performance and reduce dyspnea in PH, which may contribute to improved exercise tolerance [115].

### 6.5. Peak Oxygen Uptake and Ventilatory Efficiency

Aerobic capacity, as defined by VO2peak during CPET is an important predictor of mortality in PAH and a VO2peak below 10.4 mL/kg/min is considered a key criterion for early mortality [116,117]. In addition, ventilatory inefficiency reflected by and elevated peak VE/VCO2 and VE/VCO2 slope during CPET, where patients ventilate excessively relative to their workload [117] also contributes to exercise intolerance. In fact, VE/VCO2 slope > 60 has been shown as a strong, independent predictor of mortality in patients with PAH/CTEPH [117]. A prospective, randomized controlled trial showed significant improvements of VO2peak, cardiac index, and cardiac output during exercise, suggesting that exercise training might improve RV function in patients with PAH/CTEPH [118]. However, data on the direct effects of exercise training on ventilatory inefficiency in PH are lacking. Nevertheless, the above findings provide strong clinical evidence supporting the physiological benefits of exercise training in patients with PH.

## 7. Assessment of Exercise Capacity in Pulmonary Hypertension

Exercise training in PH enhances cardiopulmonary capacity as assessed by CPET and improves functional capacity, as evidenced by increased 6MWD. These outcomes reflect improved oxygen delivery, cardiopulmonary efficiency, and skeletal muscle conditioning [119]. Among the most widely used tools to assess the exercise capacity and to evaluate the effectiveness of exercise training in PH are the maximal incremental cardiopulmonary exercise testing (CPET), the constant load (steady state) tests, and the 6-min walking test (6MWT) [120]. These tests provide objective, quantifiable assessments of overall functional capacity and cardiopulmonary fitness, two domains significantly impaired in PH [121,122].

Cardiopulmonary exercise testing (CPET) is the gold standard for assessing exercise intolerance and provides risk stratification in PAH patients [123]. As described in the ATS/ERS statements, CPET provides a “global assessment of the integrative exercise response involving the pulmonary, cardiovascular, hematopoietic, neuropsychologic, and skeletal muscle systems that is not adequately reflected through the measurement of individual organ system function [124]. Maximal aerobic capacity, as assessed by VO2peak, is the most often reported parameter. Another important parameter assessed during the test is ventilatory threshold, which represents the point at which ventilation rises disproportionately to oxygen uptake due to the onset of anaerobic metabolism and increased lactate accumulation. In PH, this threshold typically occurs at lower workloads and in a lower percentage of VO2peak during the exercise test, reflecting impaired aerobic metabolism and reduced cardiopulmonary efficiency; these features are often more pronounced in advanced stages of the disease [120,125].

During CPET, patients with PAH typically exhibit marked hyperventilation, high ventilatory equivalents for carbon dioxide (VE/VCO2) both at ventilatory threshold and peak exercise, and low end-tidal CO_2_ (PETCO_2_), with a flat or declining PETCO_2_ response during the test [120,125,126,127,128]. Patients with combined lung and heart disease often present with a phenotype characterized by marked dyspnea and impaired gas exchange, with elevated VE/VCO_2_ slope, and abnormal ventilatory responses during exercise [119]. In mild–moderate PH with coexisting COPD, exercise is mainly limited by respiratory mechanics, whereas severe PH more closely resembles PAH with high VE/VCO_2_ slopes, hypocapnia, and circulatory limitation, suggesting that pulmonary vascular pathology mainly contributes to symptoms and exercise impairment [129].

Steady-state or constant-work-rate exercise tests are increasingly used in PR to evaluate exercise tolerance. During this test, patients exercise at a constant intensity, usually at 65–80% of the peak work rate, determined from the incremental CPET [130]. The steady state test allows cardiopulmonary variables and oxygen saturation to reach a stable level. An important variable of this test is time to the limit of tolerance (Tlim), defined as the duration a patient can sustain the constant workload until symptom-limited exhaustion. Tlim is highly sensitive to changes in exercise capacity following PR [120,131].

The 6MWT distance (6MWD) evaluates mainly submaximal exercise performance and correlates with patients’ ability to perform daily activities. The 6MWT is a widely used test in PH. Both VO2peak and 6MWD have been extensively validated as prognostic markers in PAH, and thresholds in these measures are used in current risk stratification models to guide treatment decisions [117,132,133]. Importantly, 6MWD and VO2peak have served not only as endpoints in studies evaluating exercise training but also as primary efficacy measures in clinical trials of PAH-specific pharmacotherapies [133]. Improvements in 6MWD were useful in the regulatory approval of therapies such as endothelin receptor antagonists, PDE5i, and prostacyclin analogs [134]. Similarly, VO2peak is frequently employed in research to capture the physiological impact of interventions on cardiopulmonary performance [135]. Their use in both drug development and exercise-based rehabilitation studies highlights their central role in assessing therapeutic efficacy and supports their continued inclusion in clinical trials and practice.

## 8. The Exercise Training Regimen

In most studies examining the effectiveness of exercise training in PH, the training regimen consisted of low-load cycling (continuous or intermittent, 60 s, 20–35 W), walking, light resistance exercises (weights 500–1000 g), and respiratory training, typically performed for 1.5 h per day, over 8–12 weeks, as reported in a recent meta-analysis [136]. Heart rate was generally kept below 120 bpm.

Table 3 summarizes the exercise characteristics reported in the included studies and key considerations in each study.

Various forms of PH share common clinical and hemodynamic features; however, their responses to exercise may differ and the training program needs to be adjusted accordingly. For example, Hamada et al. reported that while patients with CTEPH and idiopathic PAH had similar resting PVR, those with CTEPH exhibited a greater RV afterload both at rest and during exercise [138]. Further evaluation of the PVR–pulmonary arterial compliance relationship and the time constant of the respiratory compensation point (as evaluated by CPET) revealed important physiological differences between the groups. These findings emphasize the need for personalized therapeutic strategies tailored to the specific hemodynamic and functional characteristics of the different PH subtypes.

## 9. Practical Considerations for the Implementation of Exercise Training in PH

**Figure 1:**
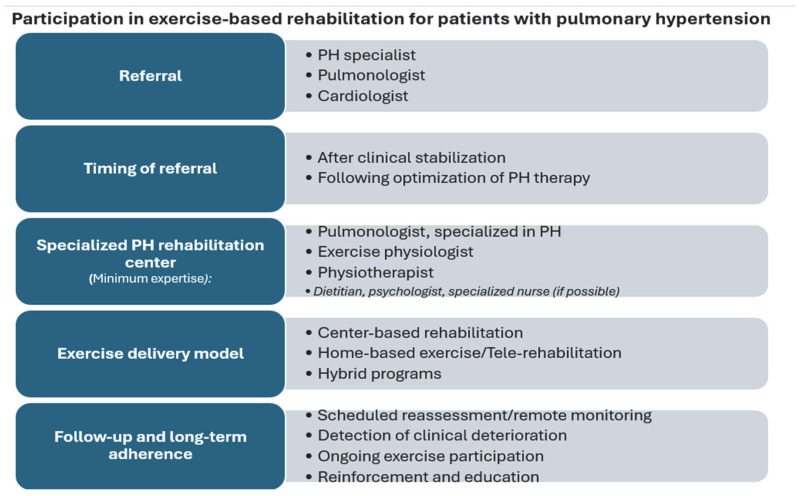
Proposed framework for implementing exercise training in pulmonary hypertension (PH), including patient selection, timing of referral, selection of the exercise setting and delivery mode, and long-term follow-up.

A structured pathway for the implementation of exercise training in PH patients has been proposed in the 2019 ERS statement [139], presenting key steps to facilitate patient participation in specialized rehabilitation programs. These include the identification of suitable patients, referral to a specialized PH rehabilitation center, supervised program implementation, and strategies to support long-term continuation of exercise in daily life. Building on this framework, a more detailed practical implementation algorithm is presented in Figure 1.

### 9.1. Exercise Setting and Delivery Mode

As shown in Table 3, in most studies exercise training was supervised, at least during the initial phases, with some programs subsequently transitioning to home-based exercise. Exclusive home-based exercise training has been evaluated only in small pilot studies with PAH and CTEPH [83]. These programs employed fully home-based or hybrid models with intermittent outpatient supervision, and training was typically performed 3–5 days per week [135]. Home-based rehabilitation was feasible and consistently improved activity-related quality of life; however, effects on maximal exercise capacity were less consistent. The smaller gains observed in some studies may reflect the lower exercise intensity and training load achievable in less supervised settings.

### 9.2. Exercise Training Implementation

**Figure 2:**
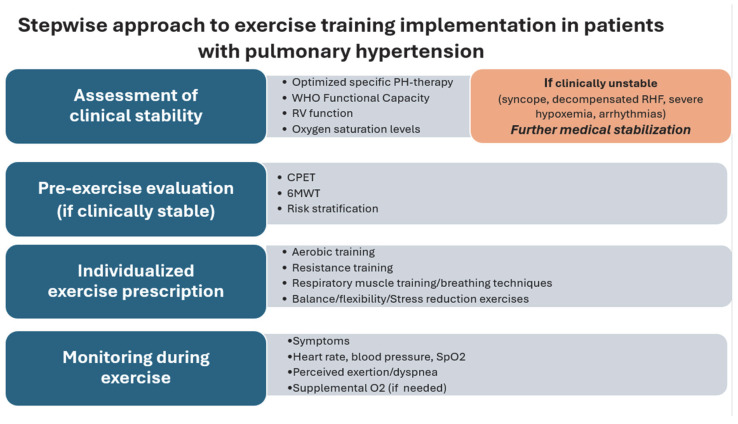
Stepwise approach to exercise training implementation in patients with pulmonary hypertension (PH).

A stepwise approach to exercise training implementation in patients with PH is presented in Figure 2.

### 9.3. Oxygen Supplementation During Exercise

Oxygen supplementation is frequently used in rehabilitation settings to prevent exertional desaturation, commonly initiated when SpO_2_ falls below 88%, although this threshold has not been specifically validated in PH populations. A systematic review in patients with precapillary PH, reported that short-term oxygen supplementation improved mean pulmonary artery pressure and exercise performance [140]. In PAH/CTEPH, oxygen supplementation prolonged submaximal exercise duration, improved ventilatory efficiency, higher cardiac output, and reduced dyspnea, likely mediated by improvements in oxygenation, cerebral oxygen delivery, and baroreceptor sensitivity [141]. Similarly, in PH associated with HFpEF without resting hypoxemia, oxygen supplementation has been shown to improve peak work rate and cycling endurance, while reducing dyspnea and VE/VCO_2_, accompanied by improved arterial oxygenation and ventilatory efficiency. A study in patients with IPF without resting hypoxemia, also showed that acute oxygen supplementation increased exercise tolerance, reduced dyspnea, and improved brain oxygenation [130], suggesting potential benefits in PH associated with ILD, although direct evidence in this population is lacking. Furthermore, another study also reported that ILD patients that were “oxygen responders,” i.e., patients that improved exercise time with oxygen supplementation, were mainly those with higher pulmonary artery pressures, supporting a possible link between pulmonary vascular involvement and oxygen responsiveness [142]. Overall, while acute benefits of supplemental oxygen on exercise performance and ventilatory efficiency are increasingly recognized across cardiorespiratory conditions, long-term studies in PH remain limited, and optimal desaturation thresholds and phenotype-specific indications for oxygen use during exercise are still unknown.

## 10. Adverse Events and Safety of Exercise

Since the first exercise training studies in PH in 2006, results have shown that carefully monitored and individually adjusted exercise training in stable PAH patients is generally safe [136]. However, some adverse events have been reported in approximately 5% of patients, most commonly including oxygen desaturation (~2.5%), and less frequently dizziness, arrhythmias, hypotension, (pre-)syncope, and fatigue [1,143]. Safety is optimized by conducting training in PH-experienced centers with pre-exercise risk stratification, exclusion of patients with recent syncope, decompensated right heart failure, uncontrolled arrhythmias, or severe resting hypoxemia, and continuous monitoring of symptoms, heart rate, blood pressure, and oxygen pulse saturation. Patients should regularly report perceived exertion and dyspnea, and exercise should be terminated if significant dyspnea occurs (Borg scale ≥ 5/10), or in the presence of dizziness, hypotension, or marked heart rate abnormalities. Adverse event management should include supervision by experienced personnel in PH rehabilitation and immediate availability of the pulmonologist in charge of clinical assessment and decision-making. Overall, serious complications have not been reported in the literature, and with appropriate supervision and individualized training protocols, the benefits of exercise rehabilitation clearly outweigh the risks.

## 11. Barriers and Enablers of Exercise Implementation in PH

Although evidence consistently supports the use of structured exercise programs for patients with pulmonary hypertension, their application in clinical settings remains inconsistent and limited. From the patient’s perspective, various symptoms and perceptions act as barriers to engagement. Common physical complaints include dyspnea, fatigue, and dizziness, all of which contribute to reduced participation in physical activity [144]. A Brazilian survey of individuals with PAH or CTEPH reported that over two-thirds of responders cited a lack of energy or motivation as major barrier to exercise [141]. Additionally, psychological distress is frequently observed in this population, and these mental health concerns often diminish patients’ confidence and willingness to engage in exercise [145]. International qualitative studies confirm that patients frequently express uncertainty and fear around physical exertion, reinforcing patterns of inactivity [146]. On the clinical side, healthcare professionals, including physicians, exercise physiologists, and physiotherapists, often report a lack of adequate training and confidence when prescribing exercise interventions specifically tailored to PH. This issue is exacerbated in non-specialized healthcare settings where rehabilitation services are rarely available. Structural limitations, such as a shortage of dedicated rehabilitation centers, poorly coordinated care systems, and insufficient healthcare funding, also play a significant role in limiting patient access to these interventions [147].

Despite these challenges, recent developments are helping to improve PR participation. In line with the 2022 ESC/ERS guidelines, exercise training is considered a reasonable and beneficial intervention for stable PAH and CTEPH patients when initiated within structured, supervised pulmonary rehabilitation programs under expert care. Following appropriate education and clinical stabilization, innovative approaches such as tele-rehabilitation have emerged as adjunct or maintenance strategies [148]. These home-based interventions enable remote monitoring, reduce the need for travel, and allow patients to exercise safely under virtual supervision, particularly benefiting those with mobility or access limitations [147]. Comparable outcomes between remote and center-based programs have been reported in both PH and chronic respiratory disease populations [149]. Furthermore, multidisciplinary care models integrating cardiologists, pulmonologists, exercise physiologists, physiotherapists, and psychologists address both medical and emotional needs, facilitating individualized exercise prescriptions and reinforcing long-term adherence [150].

These barriers and enablers can be improved through implementation of science frameworks such as the Consolidated Framework for Implementation Research (CFIR) and the RE-AIM framework (Reach, Effectiveness, Adoption, Implementation and Maintenance) that can help to structure patient-related factors (e.g., symptoms, motivation, and self-management capacity), healthcare provider readiness (e.g., knowledge, confidence, and referral behavior), and health system constraints (e.g., availability of specialized rehabilitation services and funding). In addition, patient engagement in self-monitoring and adherence, support from professional societies through guideline development and training initiatives, and facilitation by municipalities and healthcare systems through the provision of accessible rehabilitation facilities with trained personnel and structured maintenance centered or home-based exercise programs. Together, these levels influence the adoption and long-term sustainability of exercise training in PH.

Summarizing, although multiple barriers continue to limit the broader implementation of exercise training in PH, the available evidence suggests that the benefits outweigh these challenges. Interventions that combine professional education, tele-rehabilitation and wearables’ technologies, and coordinated multidisciplinary care can offer practical and effective ways to incorporate exercise into standard PH management.

## 12. Limitations and Future Directions

Despite the growing evidence supporting exercise training in PH, several limitations should be acknowledged. The available literature is dominated by relatively small studies, and most evidence derives from patients with PAH and CTEPH, whereas other PH groups remain underrepresented. Additional multicenter trials with standardized exercise interventions and longer follow-up periods are needed to evaluate the sustainability of training benefits and long-term adherence. Future research should also explore phenotype-specific exercise strategies and determine whether the mechanisms underlying exercise-induced adaptations differ across PH subtypes. Finally, studies directly comparing center-based, tele-rehabilitation, and home-based exercise programs are needed to identify the most effective and feasible models of care.

## 13. Conclusions

Exercise-based rehabilitation has moved from a once-controversial modality to an increasingly recognized component of pulmonary hypertension care. Randomized controlled trials and meta-analyses have consistently suggest that structured exercise training can improve functional capacity in clinically stable PH patients particularly in PAH and CTEPH. Proposed mechanisms include skeletal muscle and vascular adaptations and improved ventilatory efficiency, although these mechanisms require further investigation. The 2022 ESC/ERS guidelines acknowledge exercise training as a non-pharmacological intervention for selected stable patients when delivered under expert supervision. Future multicenter studies are needed to evaluate long-term outcomes, phenotype-specific strategies, and different rehabilitation modalities, including tele-rehabilitation. Overall, exercise rehabilitation appears beneficial in carefully selected, clinically stable patients managed in experienced centers, but further research is required to define its role across the full spectrum of PH.

## Figures and Tables

**Table 1:** Pulmonary hypertension classification and main etiologies.

WHO Group	Classification	Main Etiologies/Associated Conditions	Epidemiology & Clinical Relevance	Primary Pathophysiological Mechanism
Group 1	Pulmonary arterial hypertension (PAH)	Idiopathic; heritable; drug- or toxin-induced; connective tissue disease; HIV infection; portal hypertension; congenital heart disease; schistosomiasis	Rare disease (≈2–5 per million adults); female predominance; often delayed diagnosis; associated with high morbidity and mortality	Progressive remodeling of small pulmonary arteries leading to increased pulmonary vascular resistance
Group 2	PH due to left heart disease	Left ventricular systolic or diastolic dysfunction; valvular heart disease (mitral or aortic); congenital/acquired cardiomyopathies	Most common form of PH (50–85% of cases); prevalence increases with age and comorbidities; adverse prognostic impact, especially when persistent	Passive transmission of elevated left-sided filling pressures; may evolve into combined pre- and post-capillary PH
Group 3	PH due to lung diseases and/or hypoxia	Chronic obstructive pulmonary disease; interstitial lung disease; sleep-disordered breathing; alveolar hypoventilation disorders; chronic high-altitude exposure	Common in advanced lung disease; usually mild–moderate but strongly associated with reduced exercise capacity and worse survival	Hypoxic pulmonary vasoconstriction and vascular remodeling secondary to parenchymal lung disease
Group 4	Chronic thromboembolic pulmonary hypertension (CTEPH)	Incomplete resolution of acute pulmonary embolism; recurrent venous thromboembolism; thrombophilic disorders	Rare but underdiagnosed; cumulative incidence 0.5–3% after PE; only potentially curable PH subtype	Persistent mechanical obstruction of pulmonary arteries with secondary microvascular remodeling
Group 5	PH with unclear or multifactorial mechanisms	Hematologic disorders (e.g., myeloproliferative disease, sickle cell disease); metabolic disorders; chronic kidney disease; sarcoidosis; fibrosing mediastinitis; tumoral obstruction	Overall uncommon; clinically significant in specific populations; diagnosis and management require individualized assessment	Complex, heterogeneous, and often overlapping mechanisms

**Table 2:** Hemodynamic characterization of Pulmonary Hypertension (PH).

Type of Pulmonary Hypertension	Hemodynamic Features
Pre-capillary PH	mPAP > 20 mmHg
	PAWP ≤ 15 mmHg
	PVR > 2 WU
Post-capillary PH	mPAP > 20 mmHg
	PAWP > 15 mmHg
IpcPH	PVR ≤ 2 WU
CpcPH	PVR > 2 WU
Exercise PH	mPAP/CO slope > 3 mmHg/L/min between rest and exercise

PH, pulmonary hypertension; mPAP, mean pulmonary arterial pressure; PAWP, pulmonary arterial wedge pressure; PVR, pulmonary vascular resistance; IpcPH, isolated post-capillary pulmonary hypertension; CpcPH, combined post- and pre-capillary pulmonary hypertension; WU, wood units; CO, cardiac output.

**Table 3:** Exercise interventions within pulmonary rehabilitation in the different pulmonary hypertension subtypes.

Study	Pulmonary Hypertension Group/ Etiology	Participants	Exercise Intervention	Exercise Characteristics (FITT)	Main Outcomes	Key Limitations/Considerations
Mereles et al., 2006 [83]	PAH/ CTEPH	n = 30; WHO FC II–IV; stable therapy	Supervised aerobic, walking, resistance & respiratory training	15 weeks; inpatient 3 weeks → home; 60–80% peak HR	↑ 6MWD (+96 m), ↑ VO2peak, ↑ workload, ↑ QoL, improved WHO FC; no serious adverse events	Small single-center RCT; mostly PAH/CTEPH patients; limited long-term follow-up
Nagel et al., 2012 [86]	inoperable/residual CTEPH	n = 35; stable therapy	Supervised aerobic, walking, resistance & respiratory training	3 weeks inpatient + 15 weeks home; moderate intensity	↑ 6MWD (~+70 m), ↑ VO2peak, ↑ workload, ↑ QoL; ↓ NT-proBNP; no major adverse events	Small sample size; CTEPH-specific population; non-randomized design
Grünig et al., 2012 [84]	mixed etiologies: PAH, APAH, CTEPH, PH-LHD/LD	n = 183; WHO FC II–IV	Supervised aerobic, walking, resistance & respiratory training	15 weeks; inpatient 3 weeks; moderate intensity	↑ 6MWD (~+78 m), ↑ VO2peak, ↑ workload, ↑ QoL; ↓ PASP; benefits across PH groups	Observational study; heterogeneous PH population; no control group
Bussotti et al., 2017 [85]	PAH	n = 15; WHO FC II–III	Supervised outpatient aerobic + resistance & breathing exercises	4 weeks; moderate intensity	↑ 6MWD (~+8%), ↑ VO2peak, ↑ workload, ↑ O_2_ pulse, ↑ QoL; safe	Small sample size; short intervention duration
Dalla Vecchia & Bussotti, 2018 (review) [119]	PAH/CTEPH	16 studies; n = 413 WHO FC II–III	Supervised aerobic, resistance & respiratory training	3 weeks inpatient + 12 weeks home; low–moderate intensity	↑ 6MWD (~+60 m), ↑ VO2peak, ↑ QoL, improved WHO FC; exercise well tolerated	Narrative review; heterogeneous included studies and protocols
Morris et al., 2023 (Cochrane review) [135]	PAH/CTEPH)	14 RCTs; n = 462 PH mostly PAH; some CTEPH)	Supervised exercise-based rehabilitation (aerobic ± resistance)	3–25 weeks; 3–5 sessions/week; low–moderate intensity	↑ 6MWD (+48.5 m), ↑ VO2peak (+2.1 mL/kg/min), ↑ HRQoL; ↓ mPAP; no ↑ serious adverse events	limited long-term outcome data; predominantly PAH/CTEPH populations
Zeng et al., 2020 (meta-analysis) [87]	mixed etiologies: PAH, APAH, CTEPH, PH-LHD/LD	17 studies; ~650	Pooled supervised aerobic ± resistance & respiratory training	3–15+ weeks; mostly moderate intensity	↑ 6MWD (~+65 m), ↑ VO2peak (~+1.8 mL/kg/min), ↑ QoL; no serious adverse events	Substantial heterogeneity among exercise protocols and PH subtypes
Dong & Li, 2022 (systematic review) [137]	mixed etiologies: PAH, APAH, CTEPH, PH-LHD/LD	RCTs and controlled trials;	Supervised and home-based aerobic, resistance & breathing training	Variable protocols	Consistent improvements in exercise capacity, cardiopulmonary function and QoL; overall safe	Variable study quality; mixed study designs; limited data for non-PAH PH groups

PAH: Pulmonary Arterial Hypertension; APAH: Associated Pulmonary Arterial Hypertension; CTEPH: Chronic Thromboembolic Pulmonary Hypertension; PH-LHD: Pulmonary Hypertension due to Left Heart Disease; PH-LD: Pulmonary Hypertension due to Lung Disease; RCT: Randomized Controlled Trial; QoL: Quality of life; 6MWD: Increased 6-min Walk Distance; VO2peak: Peak Oxygen Uptake; FC: Improved WHO Functional Class; ↑: improvement in; ↓: reduction in.

## Data Availability

Not applicable.

## Abbreviations

The following abbreviations are used in this manuscript:

6MWT	6-min walk test
VO2peak	Peak oxygen uptake
PAH	Pulmonary arterial hypertension
COPD	Chronic obstructive pulmonary disease
ILD	Interstitial lung disease
CTEPH	Chronic thromboembolic pulmonary hypertension
PE	Pulmonary embolism
mPAP	mean pulmonary arterial pressure
PAWP	pulmonary arterial wedge pressure
PVR	pulmonary vascular resistance
IpcPH	isolated post-capillary pulmonary hypertension
CpcPH	combined post- and pre-capillary pulmonary hypertension
CO	cardiac output
